# Heightened self-reported punishment sensitivity, but no differential attention to cues signaling punishment or reward in anorexia nervosa

**DOI:** 10.1371/journal.pone.0229742

**Published:** 2020-03-03

**Authors:** Nienke C. Jonker, Klaske A. Glashouwer, Albert Hoekzema, Brian D. Ostafin, Peter J. de Jong

**Affiliations:** 1 University of Groningen, department of Clinical Psychology and Experimental Psychopathology, Groningen, The Netherlands; 2 Accare Child and Adolescent Psychiatry, Department of Eating Disorders, Groningen, The Netherlands; 3 University of Groningen, Department of Research Support, Faculty of Behavioral and Social Sciences, Groningen, The Netherlands; Harvard Medical School, UNITED STATES

## Abstract

This study examined whether adolescents with anorexia nervosa (AN) are more sensitive to punishment and less sensitive to reward than a non-eating disorder comparison group. Both self-report and performance measures were used to index reward and punishment sensitivity. Participants were adolescents with AN (*n* = 69) and an individually matched comparison group with healthy weight (*n* = 69). They completed the Behavioral Inhibition Scale/Behavioral Activation Scale and the Sensitivity to Punishment and Sensitivity to Reward Questionnaire to index self-reported reward and punishment sensitivity, and performed the Spatial Orientation Task to index attention to cues signaling reward and punishment. There was extremely strong evidence (*BF*_*10*_ > 100), that adolescents with AN reported higher sensitivity to punishment than adolescents without an eating disorder. However, adolescents with AN did not differ from the comparison group on self-reported reward sensitivity, and attention to cues signaling reward or punishment. Adolescents with AN clearly show heightened punishment sensitivity, yet this was not paralleled by a heightened proneness to detect signals of punishment. An important next step would be to examine whether punishment sensitivity is a reliable risk factor for the development or maintenance of AN.

## Introduction

Anorexia nervosa (AN) is a severe mental disorder with a high mortality rate (e.g., [1]) that typically develops during adolescence [2]. Individuals with AN’s weight and shape are overly important in their self-evaluation, they have an intense fear of gaining weight or becoming fat, and they show a striking ability to restrict their food intake even though they are often (severely) underweight [3]. The disorder is difficult to treat as many individuals do not respond to treatment, drop-out of treatment, or relapse after successful treatment [4–8]. It is therefore essential to improve our understanding of the underlying factors in the development and maintenance of AN. In the current study, we focused on the personality characteristics reward and punishment sensitivity, as these have been suggested to play an important role in AN [9].

Individuals who are sensitive to reward are thought to respond more positively to reward (e.g., hedonic response), have more attention to cues of reward, and show more approach behavior in response to cues of reward in the environment [10,11]. Individuals who are sensitive to punishment are thought to respond more negatively to punishment (e.g., aversive response), have more attention to cues of punishment, and show more avoidance behavior in response to cues of punishment in the environment [10,11]. Individuals with AN have been suggested to be sensitive to punishment (e.g., [9,12]). Behaviors such as food restriction and purging which are likely related to an intense fear of gaining weight or becoming fat (i.e., avoiding punishment), as well as the high comorbidity with anxiety disorders [13], might be the result of this relatively high punishment sensitivity. On the other hand, a relatively low sensitivity to reward might also play a role in the development and maintenance of AN. Whereas food has a high intrinsic rewarding value [14], especially when hungry [15], this might not apply to individuals with AN. For example, it has been proposed that individuals with AN might have deficits in their general sensitivity to reward, which might result in a decreased experience of food reward and might facilitate restriction of their food intake [12]. When behaviors such as food restriction are indeed the result of a general low sensitivity to reward and purging behaviors of high sensitivity to punishment, treatment might benefit from addressing these general underlying personality characteristics.

Prior studies have consistently shown that both adolescents and young adults with AN demonstrate higher self-reported sensitivity to punishment compared to participants without eating disorders [16–20]. Findings are less consistent when looking at reward sensitivity. Whereas some studies found no differences between adolescents and young adults with AN and non-eating disordered groups in self-reported reward sensitivity [16,18,20], other studies found lower sensitivity to reward [19], or even reported higher sensitivity to reward in individuals with AN [17,18].

These inconsistencies in findings might be due to the differences in questionnaires that were used [e.g., 17]. Specifically, studies reporting higher reward sensitivity in individuals with AN used the Sensitivity to Punishment and Sensitivity to Reward Questionnaire (SPSRQ [21]) [17,18], whereas studies reporting lower reward sensitivity, or no difference in reward sensitivity used the Behavioral Inhibition Scale/Behavioral Activation Scale (BIS/BAS [22]) [16,18–20]. Although the BIS/BAS and the SPSRQ are used interchangeably, the BIS/BAS is designed as a general measure of reward and punishment sensitivity [22], whereas the SPSRQ is designed to measure responses to specific situations reflecting reward or punishment [21]. Previously, it was shown that when excluding items on appearance and social rejection from the SPSRQ score, adolescents with AN scored comparable to the comparison group without an eating disorder on reward sensitivity [17]. However, this still indicates a different pattern than that of lowered reward sensitivity in individuals with AN when reward sensitivity was indexed by the BIS/BAS scale. All in all, the role of reward sensitivity in AN remains unclear. The first aim of the current study was to address these inconsistencies in findings regarding reward sensitivity by assessing a large group of adolescents with AN and a matched non-eating disordered comparison group using both the BIS/BAS and the SPSRQ to index reward and punishment sensitivity.

By including both the BIS/BAS and the SPSRQ we can limit the chance of instrument specific findings. However, it does not solve the limitations of self-report measures for which participants need self-insight and the ability to linguistically express their own tendencies to answer the questions in a meaningful way. Such expression is not easy for everyone (e.g., [23]), and might be specifically difficult for young adolescents. One way to tackle this limitation is by indexing reward and punishment sensitivity with a performance measure. Recently, a pilot study examined differences between individuals with an eating disorder and non-eating disorder comparisons in reward and punishment sensitivity with the Spatial Orientation Task (SOT) [24,25]. The SOT measures attention to cues signaling reward and punishment, which is considered a component of reward and punishment sensitivity [10,11]. More specifically, reward and punishment sensitive individuals have been suggested to be more prone to detect signals of reward and punishment, respectively [14].

In the study of Matton and colleagues [25] there was a non-significant trend that young women with an eating disorder (AN and bulimia nervosa combined) showed attentional engagement towards the cues that signal punishment compared to the non-eating disordered comparison group. No differences were found between the eating disorder group and the comparison group on attention to cues signaling reward. Thus, this earlier study provided some initial evidence for more attention to cues that signal punishment in young women with an eating disorder, but not for differential attention for cues that signal reward. However, because of the low statistical power of this study due to the small sample sizes, replication is important [25]. Therefore, the second aim of the current study was to examine differences between individuals with AN and a non-eating disordered comparison group in attention to cues that signal reward and punishment as measured with the SOT.

To sum up, the current study examined whether individuals with AN differ from a non-eating disordered comparison group in their general sensitivity for reward and punishment. Importantly, this is the first study to use both self-report measures (BIS/BAS and SPSRQ) as well as a performance measure (SOT) to index reward and punishment sensitivity. Since AN usually develops during adolescence, this study focused on adolescent patients. The following hypotheses were tested: Adolescents with AN are more sensitive for punishment and as such (1) report higher sensitivity to punishment, and (2) have more attention to cues signaling punishment; and adolescents with AN are less sensitive for reward and as such (3) report less reward sensitivity, and (4) have less attention to cues signaling reward, than a non-eating disordered comparison group.

## Materials and methods

### Participants

Patients between the ages of 12 and 23 who were referred for inpatient or outpatient treatment to the eating disorder clinic of Accare between June 2015 and June 2017, and whose primary diagnosis was AN or atypical AN according to DSM-5 criteria, were eligible to participate in the study. There were no additional in- or exclusion criteria. Participants were 69 patients (68 White, 67 female, Mean _age_ = 15.55, *SD*_age_ = 1.70), and a comparison group without an eating disorder (*n* = 69, 67 female, Mean _age_ = 15.48, *SD*_age_ = 1.82). Data of the performance measure of one participant is missing due to a computer crash during the task.

Eating disorder pathology in the patient group was examined with the child version of the Eating Disorder Examination (EDE) interview [26], and based on this, DSM-5 classifications were made. Most patients included presented with their first episode of an eating disorder (*n =* 62), and some with a second episode (*n* = 7). The patient group fulfilled the criteria of AN Restrictive type (*n* = 39), AN Binge Purge type (*n* = 10), atypical AN Restrictive type (*n* = 11), or atypical AN Binge Purge type (*n* = 9). The comparison group (CG) consisted of participants with a healthy weight who were matched on gender, age, and educational level to the patient group.

### Materials

#### Body Mass Index

Adjusted BMI was calculated ((actual BMI/Percentile 50 of BMI for age and gender) x 100) to make the BMI’s comparable over the age range [27]. The 50th percentile of BMI for age and gender was obtained from the Netherlands Organization for Applied Scientific Research [28]. Adjusted BMI scores between 85% and 120% are considered as normal weight, and smaller than 85% as underweight [29].

#### Eating disorder symptoms

The Eating Disorder Examination Questionnaire (EDE-Q [30]), was administered to assess eating disorder pathology within the past 28 days. Adaptations (comparable to adaptations that were made to the previous version of the EDE-Q [31]) were made to make the language appropriate for children and adolescents. An average score of the 22 items was used as general index of eating disorder pathology (cf. [32]). Scores can range from 0–6, and internal consistencies of this global EDE-Q score were excellent (Cronbach's alpha of .93 in patients with AN, and .95 in the comparison group).

#### Symptoms of anxiety and depression

Symptoms of anxiety and depression were assessed with the Dutch version of the Revised Child Anxiety and Depression Scale (RCADS [33]). The RCADS consists of 47 questions that can be answered on a 4-point scale ranging from *never* (0) to *always* (3). The depression subscale consists of 10 items and showed acceptable to good internal consistencies (Cronbach’s alpha of .79 in patients with AN, and of .87 in the comparison group). Anxiety was assessed by summing the items of the Social Phobia (9 items), Panic Disorder (9 items), Separation Anxiety (7 items) and Generalized Anxiety (6 items) subscales. Internal consistencies of these scores were excellent (Cronbach’s alpha of .93 in patients with AN and of .92 in the comparison group). In line with the DSM-5 categorization of anxiety disorders, the obsessive compulsive subscale was not included in the anxiety score of the current study [3].

#### Self-reported reward and punishment sensitivity

The current study included both the Behavioral Inhibition Scale/Behavioral Activation Scale (BIS/BAS [22]) and the Sensitivity to Punishment and Sensitivity to Reward Questionnaire (SPSRQ [21]) to measure self-reported reward and punishment sensitivity.

The BIS/BAS contains 24 statements, including 4 distractor items, that are answered on a 4-point scale ranging from *very false for me* (1), to *very true for me* (4). The questionnaire consists of a punishment sensitivity subscale containing 7 items (BIS; e.g., “I worry about making mistakes”), and three reward sensitivity subscales; 5 items regarding Reward Responsivity (BAS-RR; e.g., “When good things happen to me, it affects me strongly”), 4 items regarding Reward Drive (BAS-Drive; e.g., “I go out of my way to get things I want”), and 4 items regarding Fun Seeking (BAS-FS; e.g., “I crave excitement and new sensations”). The BAS-FS is not of interest in the current study, yet will be reported for the sake of completeness. Subscale scores are calculated by averaging the respective item scores. Additionally, the total reward sensitivity score (BAS-Total), which is the average of the three subscales will be reported in the descriptives. The internal consistencies of the BIS, BAS-Total, and BAS-Drive subscales in the current study were acceptable to good (Cronbach’s alpha of .78, .84, and .80, respectively in adolescents with AN, and of .83, .79, and .78 in the comparison group). The internal consistencies of the BAS-RR subscale were good in the group of adolescents with AN (Cronbach’s alpha of .80), but questionable in the comparison group (Cronbach’s alpha of .61). The internal consistencies of the BAS-FS were unacceptable to poor (Cronbach’s alpha of .49 in adolescents with AN and of .55 in the comparison group).

The SPSRQ contains 24 questions about sensitivity to reward (RS; e.g., “Do you often do things to be praised?”), and 24 about sensitivity to punishment (PS; e.g., “Are you often worried by things that you said or did?”). Participants can answer these questions with *yes* (1) or *no* (0). Subscale scores are calculated by summing the items that were answered with yes. Internal consistency of the RS was questionable to acceptable (Cronbach’s alpha of .70 in adolescents with AN and of .62 in the comparison group) and of the PS good (Cronbach’s alpha of .85 in both groups).

#### Attentional bias to general cues of reward and punishment

The Spatial Orientation Task (SOT [24]), an adaptive reaction time task, was used to measure attention towards cues of general reward and punishment [25,34]. See the Supporting information for a detailed description of the task,. The SOT indexes to what extent individuals direct their attention towards cues signaling reward and punishment (i.e., engagement), with higher scores reflecting more attentional engagement with reward and punishment respectively. It further indexes to what extent individuals have difficulty to look away from cues signaling reward and punishment (i.e., disengagement), with higher scores reflecting more difficulty to look away from reward and punishment respectively. The SOT differentiates between a more automatic process that happens within a short time period (250 ms), and a more voluntary process that happens over a somewhat longer time period (500 ms). As an estimate of the reliability of the SOT, Spearman-Brown coefficients were computed between the outcome measures for the first and the second half of the task. The relationship between these halves for all outcome measures were low with Spearman-Brown coefficients ranging from .02 to .26.

#### Procedure

This study was approved by the medical ethical committee of the University Medical Center in Groningen, the Netherlands (NL.51694042.14). Participants and their parents when they were under 18 years of age, signed informed consent forms. The Eating Disorder Examination interview and BMI assessment were part of the intake procedure at the Center for Eating Disorders, and permission was asked to use this information for the current study. Participants performed the study at the treatment center as soon as possible after intake (median 53 days after intake). Since the duration from intake to start of treatment usually takes about 4 weeks, assessment for most adolescents with AN took place at the start of treatment or up to 4 weeks after.

Participants for the comparison group were recruited at schools. For every adolescent with AN an individually matched comparison participant with a healthy weight was selected based on age and educational level. Since the International Standard Classification of Education depends highly on the number of years of education an individual has had, this classification does not seem appropriate in a sample with such a large age range as in the current sample. Participants aged 12 will have had far less years of education then participants who are 18 years old. Since all participants were still going to school, age and years of education will, in this sample, provide the same information. Therefore, we provide information about the level of education that is being followed summarized into two categories–low and high, yet the matching was done on the fine-grained level.

For adolescents with AN the study took place at the treatment center and for the comparison group at their school. Participants performed the SOT and then completed the EDE-Q, BIS/BAS, and SPSRQ. After finishing the questionnaires participants’ height and weight were measured by the researcher. The procedure for the matched controls was comparable, although they did not participate in the EDE interview. Therefore, even though we explicitly recruited adolescents without an eating disorder for the comparison group, it is unknown whether these adolescents would fulfill the DSM-5 criteria of an eating disorder. Participants and parents of both groups were informed about the content of the study with an information leaflet which was similar for both groups. All participants received 10 euros as compensation for participating in the study. The current paper reports data from a larger study on reward and punishment sensitivity (see also [35]), and the SOT was the last of five computer tasks in this study.

#### Analyses

Group differences on age, adjusted BMI, EDE-Q score, and symptoms of anxiety and depression were assessed with independent samples *t*-tests. Difference in educational level was assessed with the Chi-square test. Bivariate correlations were performed to examine the relation between the attentional bias measures and the self-report measures.

To examine whether adolescents with AN are more sensitive to punishment than the comparison group, two Multivariate Analyses of Variance (MANOVA) were performed with (1) the BIS and SP scores, and (2) the four attentional bias scores–engagement to cues signaling punishment on the short and long cue delay trials and disengagement from cues signaling punishment on the short and long cue delay trials–as dependent variables and Group (AN or comparison) as fixed factor. Univariate ANOVAs were used to examine on which variable(s) differences were found between the groups. These between subject tests had a power of 83% to find medium effects. To correct for familywise error rate a Bonferroni-Holm correction was applied. This means that for the self-report analyses the smallest *p*-value will be tested against an alpha of .025 and the largest against an alpha of .05. For the attentional bias analyses the smallest p-value will be tested against an alpha of .0125, the *p*-values following against .016 and .025, respectively, and the largest against .05.

To examine whether adolescents with AN are less sensitive for reward than the comparison group, two MANOVAs were performed with (1) the BAS-RR, BAS-Drive, BAS-Fun Seeking, and SR scores, and (2) the four attentional bias scores–engagement to cues signaling reward on the short and long cue delay trials and disengagement from cues signaling reward on the short and long cue delay trials–as dependent variables, and Group (AN or comparison) as fixed factor. Univariate ANOVAs were used to examine on which variable(s) differences were found between the groups. BAS-Total will not be included in these analyses since the three subscales already represent this score in the MANOVA. To correct for familywise error rate a Bonferroni-Holm correction was applied. This means that for both analyses, the smallest *p*-value will be tested against an alpha of .0125, the ones following against .016 and .025, respectively, and the last against .05.

Classical statistical analyses were complemented with results following the Bayesian approach to increase the confidence in our results and test the evidence for the null-hypotheses in the case of non-significant findings. Bayesian analyses were conducted with JASP [36]. Only *t*-tests were performed, since there is no option for a Bayesian MANOVA. Cauchy prior was set at the recommended default *r* = .707 [37]. We will report *BF*_*10*,_ which quantifies the evidence for the alternative hypotheses over the null hypotheses (e.g., adolescents with AN differ from the comparison group without an eating disorder in their sensitivity to reward). A Bayes factor of 1 is considered *no evidence*, between 1–3 *anecdotal*, between 3–10 *moderate*, between 10–30 *strong*, between 30–100 *very strong*, and more than 100 *extreme* evidence that the data are more likely under the alternative hypothesis. A Bayes factor between 1/3–1 is considered *anecdotal*, between 1/10–1/3 *moderate*, between 1/30–1/10 *strong*, between 1/100–1/30 *very strong*, and less than 1/100 *extremely strong* evidence that the data are more likely under null hypothesis [37].

## Results

### Group characteristics

Table 1 shows educational level, mean age, BMI, EDE-Q, anxiety and depression scores of the adolescents with AN and the comparison group without eating disorder. As expected, due to the individual matching, no differences in age and educational level were found between groups. Adolescents with AN did have a significantly lower BMI, higher scores on the EDE-Q, and higher scores on the depression and anxiety subscales of the RCADS.

**Table 1:** Group characteristics.

	CG (*n* = 69)		AN (*n* = 69)		Between-groups test
Educational level	Low	26	Low	26	*X*^*2*^ = 0.00,
	High	43	High	43	*p* = .57
	Mean	SD	Mean	SD	*t* (*p*)
Age	15.48	1.82	15.55	1.70	0.24 (.81)
BMI	102.87	9.62	84.69	12.16	-9.74 (< .001)
EDE-Q	1.30	1.10	4.16	1.11	15.17 (< .001)
Anxiety	23.22	12.35	40.59	15.10	7.40 (< .001)
Depression	7.58	4.73	15.55	4.56	10.08 (< .001)

*Note*. CG = Comparison group, AN = Patients with AN, BMI = Adjusted Body Mass Index, EDE-Q = Global score on the Eating Disorder Examination Questionnaire, Anxiety = symptoms of anxiety as measured with the Revised Child Anxiety and Depression Scale, depression = symptoms of depression as measured with the Revised Child Anxiety and Depression Scale.

### Descriptives

Table 2 shows the attentional bias scores and the mean scores on the self-report measures for the group of adolescents with AN and the comparison group. Bivariate correlations between the measures of reward and punishment sensitivity can be found in Table 3. Importantly, age and educational level were related to some of the reward and punishment sensitivity measures, and were therefore included as covariates in the analyses. Age was related to BAS-Drive (*r* = 0.18, *p* = .033), RS (*r* = 0.22, *p* = .010), and reward engagement short (*r* = 0.20, *p* = .019). Educational level was related to BIS (*r*_*s*_ = 0.17, *p* = .045), punishment disengagement short (*r*_*s*_ = -0.23, *p* = .008), reward engagement short (*r* = -0.18, *p* = .039), and reward disengagement long (*r*_*s*_ = 0.20, *p* = .020). Anxiety and depression were not included as covariates, since anxiety and depression are known characteristics of individuals with AN [cf., 38]. This is also reflected by the strong relationship between eating disorder symptoms as measures with the EDE-Q and symptoms of anxiety (*r* = 0.65, *p* < .001) and depression (*r* = 0.78, *p* < .001), and the moderate relationship between BMI and symptoms of anxiety (*r* = -0.31, *p* < .001) and depression (*r* = -0.33, *p* < .001). Importantly, statistically controlling for known pre-existing group differences likely results in uninterpretable results [38].

**Table 2:** Mean scores of reward and punishment sensitivity per group.

	CG		AN	
	(*n* = 69^1^)		(*n* = 69)	
	*Mean*	*SD*	*Mean*	*SD*
**Punishment sensitivity**				
BIS	2.83	0.57	3.30	0.53
PS	10.90	5.40	15.87	5.02
Punishment engagement 250ms	-43.72	38.21	-33.26	37.66
Punishment engagement 500 ms	-33.92	58.24	-8.62	67.04
Punishment disengagement 250 ms	35.54	90.99	24.89	79.50
Punishment disengagement 500 ms	4.96	88.06	-2.70	70.31
**Reward sensitivity**				
BAS-RR	3.22	0.37	3.07	0.58
BAS-Drive	2.63	0.58	2.51	0.68
BAS-FS	2.85	0.52	2.62	0.52
BAS-Total	2.92	0.38	2.76	0.48
RS	11.52	3.39	10.48	3.71
Reward engagement 250 ms	40.84	38.59	39.42	59.61
Reward engagement 500 ms	33.73	58.43	30.69	57.28
Reward disengagement 250 ms	-4.74	73.32	-23.50	72.80
Reward disengagement 500 ms	-12.99	66.32	11.17	68.07

Note.

^1^ Attention to cues signaling reward and punishment is reported of 68 participants. CG = Comparison group, AN = Adolescents with AN, BIS = punishment sensitivity of the BIS/BAS, PS = Punishment Sensitivity of the SPSRQ, BAS-RR = Reward responsivity of the BIS/BAS, BAS-Dr = Reward drive of the BIS/BAS, BAS-FS = Fun Seeking of the BIS/BAS, RS = Reward Sensitivity of the SPSRQ.

**Table 3:** Bivariate correlations between measures of reward and punishment sensitivity.

		Punishment						Reward							
		1.	2.	3.	4.	5.	6.	7.	8.	9.	10.	11.	12.	13.	14.
Punishment	1. BIS	-	-	-	-	-	-	-	-	-	-	-	-	-	-
	2. PS	.72**	-	-	-	-	-	-	-	-	-	-	-	-	-
	3. Punishment engagement 250ms	0.10	.05	-	-	-	-	-	-	-	-	-	-	-	-
	4. Punishment engagement 500 ms	.27**	-19*	.08	-	-	-	-	-	-	-	-	-	-	-
	5. Punishment disengagement 250 ms	-.14	-.11	-.28**	.07	-	-	-	-	-	-	-	-	-	-
	6. Punishment disengagement 500 ms	-.14	-.13	-.10	.11	-.01	-	-	-	-	-	-	-	-	-
Reward	7. BAS-RR	.01	-.13	.05	.07	.01	-.02	-	-	-	-	-	-	-	-
	8. BAS-Drive	-.20*	-.18*	.00	.01	.06	.05	.47**	-	-	-	-	-	-	-
	9. BAS-FS	-.21*	-.30**	.03	.02	-.04	.05	.55**	.37**	-	-	-	-	-	-
	10. BAS-Total	-.16	-.25**	.03	.04	.02	.03	.85**	.79**	.77**	-	-	-	-	-
	11. RS	-.13	-.15	-.02	.00	.01	.11	.45**	.46**	.40**	.55**	-	-	-	-
	12. Reward engagement 250 ms	-.14	-.03	-.41**	-.12	.36**	.17*	-.16	.10	-.10	-.06	-.02	-	-	-
	13. Reward engagement 500 ms	-.02	.05	-.03	-.08	-.12	-.04	-.20*	-.05	-.11	-.15	-.09	.13	-	-
	14. Reward disengagement 250 ms	-.11	-.08	.14	-.02	-.12	-.19	.07	-.04	-.02	.01	.02	-.26**	-.01	-
	15. Reward disengagement 500 ms	.13	.22**	-.05	.14	-.05	-.19	.07	-.02	-.04	.01	-.02	-.06	.04	-.04

Note.

* *p* < .05

** *p* < .01. BIS = punishment sensitivity of the BIS/BAS, PS = Punishment Sensitivity of the SPSRQ, BAS-RR = Reward responsivity of the BIS/BAS, BAS-Dr = Reward drive of the BIS/BAS, BAS-FS = Fun Seeking of the BIS/BAS, RS = Reward Sensitivity of the SPSRQ.

### Are adolescents with AN more sensitive to punishment than the comparison group?

#### Self-report

A significant difference was found between adolescents with AN and the comparison group on self-reported punishment sensitivity (*Λ* = 0.79, *F*(2,133) = 17.65, *p* < .001, *η*^*2*^_*p*_ = 0.21, *CI* [0.11, 0.30]). Between subjects test showed that adolescents with AN scored higher than the comparison group on both questionnaire measures, and the Bayesian analyses shows extremely strong evidence that the current data are more likely under the alternative hypothesis that adolescents with AN are more sensitive to punishment than adolescents without an eating disorder (BIS: *α* = .05, *F*(1,134) = 26.12, *p* < .001, *η*^*2*^_*p*_ = 0.16, *CI* [0.08, 0.26], *BF*_*10*_ BIS = 17615; PS: *α* = .025, *F*(1,134) = 32.16, *p* < .001, *η*^*2*^_*p*_ = 0.19, *CI* [0.11, 0.29], *BF*_*10*_ PS = 210447).

#### Attentional bias

Adolescents with AN did not differ from the non-eating disorder comparison group in their attentional bias for cues signaling punishment (*Λ* = 0.94, *F*(4,130) = 2.11, *p* = .08, *η*^*2*^_*p*_ = 0.06, *CI* [0.00, 0.11]). Bayesian independent samples *t*-tests showed moderate evidence that the observed data regarding disengagement from cues signaling punishment on the short (*BF*_*10*_ = 0.11) and long (*BF*_*10*_ = 0.13) cue delay trials are more likely under the null hypothesis that adolescents with AN do not have more disengagement from cues signaling punishment than adolescents without an eating disorder. The analyses show anecdotal evidence that the observed data regarding engagement to cues signaling punishment on the short cue delay trials (*BF*_*10*_ = 1.12), and moderate evidence that the observed data regarding the long cue delay trials (*BF*_*10*_ = 4.43) are more likely under the alternative hypothesis that adolescents with AN have more engagement to cues signaling punishment than adolescents without an eating disorder.

### Are adolescents with AN less sensitive to reward than the comparison group?

*Self-report*. Adolescents with AN did not differ from the comparison group on self-reported sensitivity to reward (*Λ* = 0.95, *F*(4,131) = 1.82, *p* = .13, *η*^*2*^_*p*_ = 0.05, *CI* [0.00, 0.10]). Bayesian independent samples *t*-tests showed anecdotal evidence that the observed data on BAS-Drive (*BF*_*10*_ = 0.55) are more likely under the null hypothesis that adolescents with AN do not differ from adolescents without an eating disorder in BAS-Drive. The analyses showed anecdotal to moderate evidence that the observed data on BAS-RR (*BF*_*10*_ = 1.39), BAS-FS (*BF*_*10*_ = 6.87), and RS (*BF*_*10*_ = 1.34) are more likely under the alternative hypothesis that adolescents with AN are less sensitive to reward than adolescents without an eating disorder.

*Attentional bias*. Adolescents with AN did not differ from the comparison group in their attentional bias for cues signaling reward (*Λ* = 0.95, *F*(4,130) = 1.72, *p* = .15, *η*^*2*^_*p*_ = 0.05, *CI* [0.00, 0.10]). Bayesian independent samples t-tests showed anecdotal to strong evidence that the observed data on engagement to cues signaling reward on the short (*BF*_*10*_ = 0.21) and long (*BF*_*10*_ = 0.24) cue delay trials, and disengagement from cues signaling reward on the short (*BF*_*10*_ = 0.94) and long cue delay time trials (*BF*_*10*_ = 0.06) are more likely under the null hypotheses that adolescents with AN do not differ from adolescents without an eating disorder in their attention for cues signaling reward.

### Post-hoc analyses

To follow-up on the finding that adolescents with AN are more sensitive to punishment than adolescents without an eating disorder, we examined the relationship between sensitivity to punishment and BMI, eating disorder symptoms, and symptoms of anxiety and depression. BIS was significantly negatively related to BMI (*r* = -0.33, *p* < .001), and positively to eating disorder symptoms (*r* = 0.33, *p* < .001), symptoms of anxiety (*r* = 0.67, *p* < .001), and symptoms of depression (*r* = 0.40, *p* < .001). PS was significantly negatively related to BMI (*r* = -0.34, *p* < .001), and positively to eating disorder symptoms (*r* = 0.42, *p* < .001), symptoms of anxiety (*r* = 0.76, *p* < .001), and symptoms of depression (*r* = 0.55, *p* < .001).

Additionally, we examined to what extent eating disorder symptoms as measured with BMI or the EDE-Q are independently related to punishment sensitivity, over and above symptoms of anxiety and depression. Therefore, hierarchical regression models were tested with BIS and PS as dependent variables. All independent variables were centered before being entered into the models. Both in the model with BIS and the model with PS as dependent variable, BMI (BIS: *β* = -.16, *t* = -2.49, *p* = .014; PS: *β* = -.12, *t* = -2.09, *p* = .039) showed an independent negative relationship with punishment sensitivity when symptoms of anxiety (BIS: *β* = .87, *t* = 9.03, *p* < .001; PS: *β* = .82, *t* = 9.52, *p* < .001) and depression (BIS: *β* = -.24, *t* = -2.05, *p* = .042; PS: *β* = -.01, *t* = 0.07, *p* = .946) were also included in the model. Thus, higher punishment sensitivity was related to a lower BMI over and above symptoms of anxiety and depression. Eating disorder symptoms as measured with the EDE-Q were not independently related to punishment sensitivity (BIS: *β* = -.06, *t* = -0.55, *p* = .581; PS: *β* = -.12, *t* = -1.33, *p* = .187) over and above symptoms of anxiety (BIS: *β* = -.89, *t* = -9.03, *p* < .001; PS: *β* = .84, *t* = 9.53, *p* < .001) and symptoms of depression (BIS: *β* = -.25, *t* = -2.05, *p* = .043; PS: *β* < .001, *t* = 0.03, *p* = .979). Thus, punishment sensitivity was not related to self-reported eating disorder symptoms over and above symptoms of anxiety and depression.

The analyses on differences between adolescents with AN and non-eating disordered comparisons were also performed excluding healthy controls who scored relatively high on the eating disorder examination questionnaire (2.3 or higher; 38) from the comparison group (*n* = 17). Although these adolescents in the comparison group were not in treatment for an eating disorder and they had a healthy weight, they could be considered symptomatic. However, outcomes of the four MANOVAs were comparable with the analyses including all participants, and resulted in the same conclusions (self-reported punishment sensitivity: *Λ* = 0.75, *F*(2,119) = 19.48, *p* < .001, *η*^*2*^_*p*_ = 0.25, *CI* [0.13, 0.34]; attention to cues signaling punishment: *Λ* = 0.94, *F*(4,117) = 1.76, *p* = .141, *η*^*2*^_*p*_ = 0.06, *CI* [0.00, 0.11]; self-reported reward sensitivity: *Λ* = 0.95, *F*(4,117) = 1.43, *p* = .228, *η*^*2*^_*p*_ = 0.05, *CI* [0.00, 0.09]; attention to cues signaling punishment and cues signaling reward sensitivity: *Λ* = 0.95, *F*(4,117) = 1.63, *p* = .170, *η*^*2*^_*p*_ = 0.05, *CI* [0.00, 0.10].

We performed post-hoc analyses to examine whether there are differences between adolescents with AN-R and AN-BP in their reward and punishment sensitivity. Table 4 provides the means for the AN-R and AN-BP groups separately. AN-R patients had a lower educational level, a lower BMI and less symptoms of depression than AN-BP patients. Additionally, AN-R patients had marginally lower EDE-Q scores than AN-BP patients. Our exploratory analyses on differences between the subtypes of AN showed a significant difference between AN-R and AN-BP patients on self-reported punishment sensitivity (*Λ* = 0.87, *F*(2,66) = 5.11, *p =* .009, *η*^*2*^_*p*_ = 0.13, *CI* [0.2, 0.25]). Between subjects tests showed that the AN-R group scored higher on both BIS (*F*(1,67) = 9.39, *p* = .003, *η*^*2*^_*p*_ = 0.12, *CI* [0.03, 0.25], *BF*_*10*_ = 11.87) and PS (*F*(1,67) = 7.10, *p* = .01, *η*^*2*^_*p*_ = 0.10, *CI* [0.01, 0.21], *BF*_*10*_ = 4.85) than the AN-BP group. There was no evidence for a difference in attention for cues signaling punishment between patients with AN-R and AN-BP (*Λ* = 0.91, *F*(4,64) = 1.55, *p* = .20, *η*^*2*^_*p*_ = 0.09, *CI* [0.00, 0.16]). Bayesian independent samples *t*-tests showed anecdotal to moderate evidence that the observed data on engagement to cues signaling punishment on the short (*BF*_*01*_ = 3.67) and long (*BF*_*01*_ = 3.33), and disengagement from cues signaling punishment on the short (*BF*_*01*_ = 1.12) and long (*BF*_*01*_ = 1.12) cue delay trials are more likely under the null hypothesis that the groups do not differ in attention to cues signaling punishment.

The exploratory analyses showed no significant difference between AN-R and AN-BP patients in their self-reported reward sensitivity (*Λ* = 0.88, *F*(4,64) = 2.26, *p* = .08, *η*^*2*^_*p*_ = 0.12, *CI* [0.00, 0.21]). Bayesian independent samples t-tests showed anecdotal to moderate evidence that the observed data on BAS-RR (*BF*_*01*_ = 1.30), BAS-Drive (*BF*_*01*_ = 3.67), BAS-FS (*BF*_*01*_ = 2.94), and RS (*BF*_*01*_ = 2.41) are more likely under the null hypothesis that individuals with AN-R and AN-BP do not differ in self-reported reward sensitivity. Additionally, no differences between patients with AN-R and AN-BP were found on attentional bias to cues signaling reward (*Λ* = 0.93, *F*(4,64) = 1.16, *p* = .34, *η*^*2*^_*p*_ = 0.07, *CI* [0.00, 0.13]). Bayesian independent samples t-tests showed anecdotal to moderate evidence that the observed data on engagement to cues signaling reward on the short (*BF*_*01*_ = 1.00) and long (*BF*_*01*_ = 2.49), and disengagement from cues signaling reward on the short (*BF*_*01*_ = 1.48) and long (*BF*_*01*_ = 3.27) cue delay trials are more likely under the null hypothesis that there is no difference in attention to cues signaling reward between individuals with AN-R and AN-BP.

**Table 4:** Group characteristics of patients with AN-R and AN-BP.

	AN-R (*n* = 50)		AN-BP (*n* = 19)		Between-diagnosis test	
Educational level	Low	14	Low	12	*Χ*^*2*^ = 7.25,	
	High	36	High	7	*p* < .01	
	*Mean*	*SD*	*Mean*	*SD*	*t*	*p*
Age	15.42	1.55	15.89	2.05	-1.04	.304
BMI	82.72	10.86	89.87	14.10	-2.24	.028
EDE-Q	4.01	1.14	4.57	0.96	-1.90	.062
Anxiety	40.96	14.67	39.63	16.57	0.32	.747
Depression	14.84	4.55	17.42	4.14	-2.16	.035
**Punishment sensitivity**						
BIS	3.41	0.47	3.00	0.59		
PS	16.82	4.29	13.37	5.98		
Punishment engagement 250ms	-33.02	37.56	-33.89	38.94		
Punishment engagement 500 ms	-6.15	62.02	-15.11	80.30		
Punishment disengagement 250 ms	14.99	70.95	50.95	95.85		
Punishment disengagement 500 ms	-11.44	64.47	20.33	81.18		
**Reward sensitivity**						
BAS-RR	3.15	0.55	2.89	0.62		
BAS-Drive	2.52	0.69	2.50	0.68		
BAS-FS	2.60	0.51	2.70	0.54		
BAS-Total	2.78	0.46	2.71	0.55		
RS	10.20	3.76	11.21	3.58		
Reward engagement 250 ms	31.68	35.33	59.78	97.05		
Reward engagement 500 ms	26.55	55.61	41.57	61.69		
Reward disengagement 250 ms	-15.53	69.48	-44.46	78.99		
Reward disengagement 500 ms	13.88	75.10	4.03	45.57		

*Note*. BMI = Adjusted Body Mass Index, EDE-Q = Total score on the Eating Disorder Examination Questionnaire, Anxiety = symptoms of anxiety as measured with the Revised Child Anxiety and Depression Scale, depression = symptoms of depression as measured with the Revised Child Anxiety and Depression Scale, AN-R = Adolescents with AN restrictive subtype, AN-BP = Adolescents with AN binge purging subtype, BIS = punishment sensitivity of the BIS/BAS, PS = Punishment Sensitivity of the SPSRQ, BAS-RR = Reward responsivity of the BIS/BAS, BAS-Dr = Reward drive of the BIS/BAS, BAS-FS = Fun Seeking of the BIS/BAS, RS = Reward Sensitivity of the SPSRQ.

## Discussion

The current study set out to examine whether adolescents with AN differed from those without an eating disorder in their general sensitivity to reward and punishment. This is the first study to use both self-report as well as a performance measure to index reward and punishment sensitivity in AN. Furthermore, it is the first study on reward and punishment sensitivity in AN to use Bayesian statistics, providing the opportunity to quantify the evidence in favor of the null hypothesis. The main findings can be summarized as follows: Adolescents with AN (1) reported higher sensitivity to punishment as measured with both the BIS/BAS and SPSRQ; (2) did not show more attention to cues signaling punishment; (3) did not report lower sensitivity to reward as measured with the BIS/BAS and SPSRQ; and (4) did not show less attention to cues signaling rewarding, than adolescents without an eating disorder.

In line with previous findings, adolescents with AN in the current study reported higher sensitivity for punishment than adolescents without an eating disorder [16–20]. This difference was found when assessing punishment sensitivity with the BIS/BAS as well as with the SPSRQ. In the current study, there was extremely strong evidence in favor of a difference between the groups (*BF*_*10*_ > 100) for both questionnaires. All in all, the finding that individuals with AN report a higher sensitivity to punishment than non-eating disordered comparisons seems robust. Furthermore, post-hoc analyses showed that punishment sensitivity might be higher in individuals with AN-R than in individuals with AN-BP. Bayesian analyses showed moderate to strong evidence in favor of this group difference. Nevertheless, this finding should be replicated since the AN-BP group consisted of a very limited number of individuals (*n* = 19). Future studies should further examine whether this relatively high punishment sensitivity plays a role in the development and/or maintenance of the disorder, whether it is a personality characteristic that fluctuates together with AN or whether high reports of punishment sensitivity is a consequence of the disorder. Previously, punishment sensitivity was not found to relate to persistence of eating disorder symptoms [17], and treatment of AN was not found to result in a decrease in punishment sensitivity [39]. However, in these studies the change in punishment sensitivity was not examined in relationship to change in eating disorder symptoms, and symptoms of anxiety and depression.

Although adolescents with AN reported higher punishment sensitivity than the comparison group, there was no clear evidence for a difference in attention to cues signaling punishment between the two groups. The Bayesian analyses showed that there was moderate evidence that there was no difference in disengagement from general cues signaling punishment on either the short or long cue delay trials, and the evidence for the engagement to cues signaling punishment was inconclusive for the short cue delay trails. There was moderate evidence for a difference in engagement to cues signaling punishment on the long cue delay trails. This finding seems to be in contrast with the only previous study looking at attention to general cues signaling punishment in which no difference on attentional engagement was found [25]. Furthermore, since the overall analyses did not reach statistical significance whereas the current study had substantial power the most tenable conclusion is that there is no clear difference in attention to cues signaling punishment between individuals with AN and individuals without an eating disorder.

The discrepancy between the results of the self-report and performance measures might indicate that these measures tap into different aspects of punishment sensitivity. First of all, this might imply that the relatively high sensitivity to punishment as reported by individuals with AN is limited to their own experience, but is not reflected in their actual behavior. Alternatively, it might be that attention is not the most relevant behavior related to punishment sensitivity in the context of AN. It has been suggested that individuals who are sensitive to punishment respond more negatively to punishment, have more attention to punishment, and show more avoidance behavior in response to punishment in the environment [10,11]. The self-report measures of punishment sensitivity, the BIS/BAS and the SPSRQ, on which individuals with AN score relatively high, mainly seem to index punishment responsivity (e.g., BIS/BAS: “Criticism or scolding hurts me quite a bit”), and punishment avoidance (e.g., SPSRQ: “Do you often refrain from doing something you like in order not to be rejected or disapproved of by others?”). It might thus be that individuals with AN do not differ in attention to cues signaling punishment, but would differ on behavioral measures that index responsivity or avoidance behavior.

Post-hoc regression analyses showed that when anxiety and depression symptoms were statistically controlled, BMI was still significantly related to punishment sensitivity. Thus, the relationship between BMI and punishment sensitivity was partly independent of symptoms of anxiety and depression. However, the relationship between EDE-Q scores and punishment sensitivity was no longer significant after including anxiety and depression symptoms in the model. Thus, our post-hoc analyses seem to indicate that heightened PS scores in individuals with AN might, at least partly, reflect their heightened symptoms of anxiety. Since AN often co-occurs with symptoms of depression and anxiety (e.g., [40]) which was also the case in the current sample, and considering that punishment sensitivity has been suggested to be a risk factor for developing symptoms of anxiety and depression (e.g., [10,41]), heightened punishment sensitivity might contribute to the development of anxiety symptoms in individuals with AN. Future studies examining the role of punishment sensitivity in the development and/or maintenance of AN might thus also want to incorporate the relationship with symptoms of anxiety and depression in individuals with AN.

The current study did not find a difference between adolescents with AN and a non-eating disordered comparison group in self-reported reward sensitivity as measured with the BIS/BAS and the SPSRQ. Not finding a difference between adolescents with AN and a comparison group in self-reported reward sensitivity as measured with the BIS/BAS is in line with most studies [16,18,20], yet inconsistent with one study [19]. However, when examining the latter study more closely, it appears that in that study, the lower reward sensitivity in patients with AN is due to lower reports on BAS fun seeking specifically. Indeed, in the current study the Bayesian analysis showed moderate evidence for lower BAS fun seeking in patients with AN, yet only anecdotal evidence for lower reward responsivity or reward sensitivity as measured with the SPSRQ However, since the BAS fun seeking subscale has been suggested to measure impulsivity or sensation seeking rather than reward sensitivity [42], it seems reasonable to conclude that the studies using the BIS/BAS consistently find no differences between individuals with AN and a comparison group in self-reported reward sensitivity. The absence of a difference between adolescents with AN and a comparison group in self-reported reward sensitivity as measured with the SPSRQ is inconsistent with previous findings [17,18]. Interestingly, the difference in findings seems to result from differences in reported reward sensitivity in the comparison group (mean of 8.05 in Glashouwer et al. [17] vs 11.52 in the current study), while reward sensitivity as reported by individuals with AN seems comparable (mean of 9.70 in Glashouwer et al. [17] vs. 10.48 in the current study). Unfortunately, the scores of the study of Jappe et al. [18] cannot be compared since they used an adapted subscale. In addition, findings on reward sensitivity as measured with the SPSRQ should be interpreted with caution since they might not reflect general reward sensitivity, but a sensitivity to the specific situations that are asked about in the questionnaire [17].

The current study also failed to find differences in attention to cues signaling reward between adolescents with AN and the comparison group. This is in line with a pilot study that showed no differences between eating disorder individuals and a comparison group on attention to cues signaling reward as measured with the same task [25]. Findings of the current study are also in line with an fMRI study showing no difference in brain activation in response to reward anticipation between individuals with AN and healthy women [43]. All in all, findings mostly seem to indicate that there is no difference between individuals with AN and non-eating disordered comparisons in reward sensitivity. Furthermore, post-hoc analyses also did not provide evidence for a difference in reward sensitivity between individuals with AN-R versus those with AN-BP on either self-report or attentional bias.

The current study has several strengths, such as the large group of individuals with AN and the individually matched comparison group. Additionally, a behavioral measure of reward and punishment sensitivity that assesses attentional bias to cues that signal reward and punishment was included. Nevertheless, the current study also has some limitations that should be taken into account when interpreting the results. First, estimates of reliability in terms of spilt-half reliability coefficients for the SOT were low. Yet, this should be interpreted with some caution since the indices used in the split-half reliability analyses are calculated from less trials than are expected to be necessary for an acceptable signal to noise ratio. That means that two a priori unreliable indices are compared in the split-half reliability analyses [44]. Second, even though we specifically recruited adolescents without eating problems for the comparison group, this was not checked with a diagnostic interview. However, the EDE-Q was used to assess eating disorder symptoms and a healthy BMI was required. It is therefore very unlikely that participants in the comparison group had (substantial) eating problems.

## Conclusions

To conclude, the current study did not find any evidence for a difference in reward sensitivity between adolescents with AN and a non-eating disordered comparison group. However, the current study did show that adolescents with AN reported higher sensitivity to punishment than adolescents without an eating disorder. This finding seems robust since it is consistently found also in previous studies, and occurred regardless of the questionnaire that was used to assess punishment sensitivity. It would be important for future studies to examine the specific role of heightened punishment sensitivity in the development and persistence of AN. When high punishment sensitivity is related to symptom persistence, treatment might benefit from addressing this general sensitivity to punishment.

## Supporting information

S1 Detailed Description of the Spatial Orientation Task. (DOCX)
