# Supplementary material for: Heightened self-reported punishment sensitivity, but no differential attention to cues signaling punishment or reward in anorexia nervosa
Source: PLoS One. 2020 Mar 3;15(3):e0229742. doi: 10.1371/journal.pone.0229742 (PMC7053765; doi:10.1371/journal.pone.0229742)
Supplement: S1 Detailed — (DOCX) [file pone.0229742.s001.docx]

**S1 Detailed description of the Spatial Orientation Task**

The SOT was completed on a HP Probook 650 G1 running Windows 7 on a 15-inch monitor (1366 x 768 pixels). Screen refresh rate was set at 60 Hz, and the task was programmed in E-prime 2.0 [1]. Participants were seated 50 cm away from the screen and responses were collected with a response box with two buttons. The response box was placed in front of the participants with the buttons arranged vertically and participants were told that they only needed to use to button closest to them, although both buttons worked.

The following were displayed throughout the task (see S1 Fig for an example of a trial): the current score in the middle of the screen; and two small black bars, one on the right and one on the left side of the score. Participants were instructed to pay attention to this score during the game. At the start of each trial the score disappeared from the screen for 200 ms; 250 ms after the score reappeared, a cue replaced one of the two black bars. This cue was either a blue arrow pointing upward, or a red arrow pointing downward. After either 250 ms (short delay) or 500 ms (long delay), a small grey rectangle (the target) appeared within the cue (cued trial), or within the remaining black bar (uncued trial). Participants were instructed to respond with a button press on the response box as soon as they saw the target. Two thirds of the targets appeared in the cued location. The blue cue signaled that responding to the cued target would be easy and it results in a fast enough response 75% of the time. Responding to the uncued target in a blue cue trial would be hard and results in an insufficiently fast response 75% of the time. For the red cue it is the opposite, responding to the cued target would be hard and it results in an insufficiently fast response 75% of the time. Responding to an uncued target in a red cue trial would be easy and results in a fast enough response 75% of the time. Thus, in general the blue cue was a signal for a high chance of a fast enough response, and the red cue a signal for a high chance of a too slow response. Participants were informed about this difference between the cues in the instructions. In some trials no target appeared (catch trials), and for those trials, participants were instructed to not press the button. At the end of each trial a feedback signal was presented in the middle of the screen directly below the score. Here again a blue or red arrow was used. The blue arrow pointing upwards signaled a fast enough response on targeted trials or a correct nonresponse on catch trials. The red arrow pointing downward signaled a too slow response on targeted trials, a response before the target appeared on targeted trials, or an inappropriate response on catch trials. 250 ms after the feedback signal appeared, the score in the middle of the screen was changed. There was a random inter trial interval of either 500 or 1000 ms.

The task consisted of two different types of blocks (games). During winning games, participants would win 10 points on each trial that they responded sufficiently fast on, and would not win points when they responded too slowly. During losing games, participants would lose 10 points when they responded too slowly and would not lose points when they responded sufficiently fast on a trial. Regardless of the block, participants would lose 10 points if they responded inaccurately (i.e., before the target was shown or on catch trials). The task started with an instruction block, with 7 cued, 6 uncued and 1 catch trial, all trials with a long delay after the cue. This instruction block was followed by two practice blocks – a winning and a losing – each consisting of 6 cued, 6 uncued, and 2 catch trials. After the practice blocks, all participants started the test with two winning games, continued with two losing games followed by another two winning and two losing games. Each game consisted of 32 cued trials (57%), 16 uncued trials (29%) and 8 catch trials (14%) in random order.

| 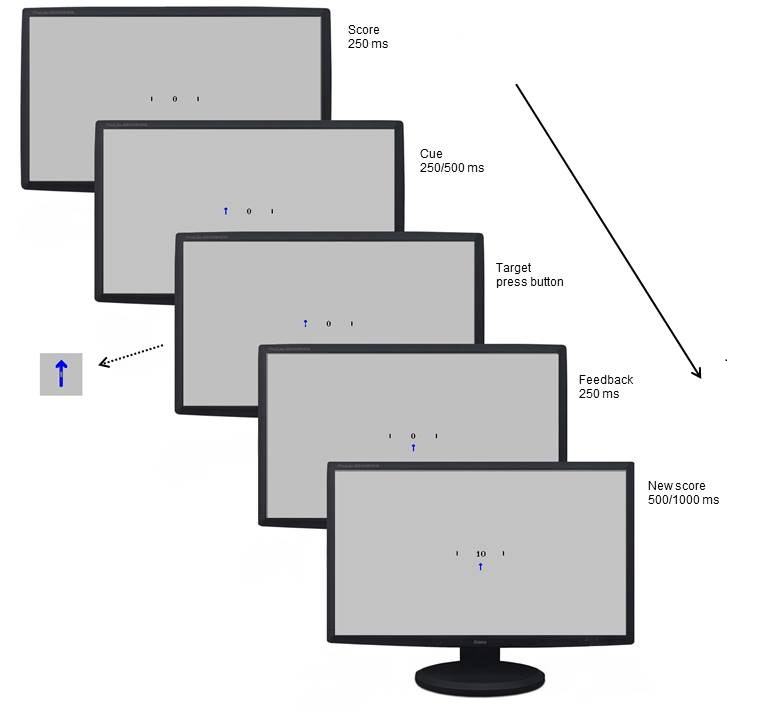 |
| --- |
| *S1 Fig.* Example of a blue cue, cued trial with a sufficiently fast response in a winning game. |

At the end of each game the participant’s median reaction time and standard deviation were calculated to compute cutoffs for fast and slow responses in the following game of the same type. For the three practice blocks, a fixed cutoff of 350 ms was used since no personalized cutoffs were available for these blocks. During easy trials (cued blue or uncued red) responses were labeled sufficiently fast when they were faster than participant’s median reaction time plus 0.55 times the standard deviation. During hard trials (uncued blue or cued red) responses were labeled sufficiently fast when they were faster than participant’s median reaction time minus 0.55 times the standard deviation. Further, since reaction times tend to be about 25 ms slower after a short cue delay time then after a long cue delay time [2], 12 ms were added to the median reaction time for short-delay trials and 12 ms were subtracted from the median reaction time for long-delay trials (See S1 Table for an overview).

| S1 Table. *Overview of trials of the spatial orientation task* | | | | | | |
| --- | --- | --- | --- | --- | --- | --- |
| **Cue** | **Target** | **Odds** | **Cue delay time** | **Cutoff for fast response^1^** | **Correction for cue delay time** | **Anticipated outcome** |
| Blue | Cued | 2/3 | 250 ms | Median RT **+** 0.55 SD | **+** 12 ms | 75% chance of **positive** outcome |
|  | Cued | 2/3 | 500 ms | Median RT **+** 0.55 SD | **–** 12 ms | 75% chance of **positive** outcome |
|  | Uncued | 1/3 | 250 ms | Median RT **–** 0.55 SD | **+** 12 ms | 75% chance of **negative** outcome |
|  | Uncued | 1/3 | 500 ms | Median RT **–** 0.55 SD | **–** 12 ms | 75% chance of **negative** outcome |
| Red | Cued | 2/3 | 250 ms | Median RT **–** 0.55 SD | **+** 12 ms | 75% chance of **negative** outcome |
|  | Cued | 2/3 | 500 ms | Median RT **–** 0.55 SD | **–** 12 ms | 75% chance of **negative** outcome |
|  | Uncued | 1/3 | 250 ms | Median RT **+** 0.55 SD | **+** 12 ms | 75% chance of **positive** outcome |
|  | Uncued | 1/3 | 500 ms | Median RT **+** 0.55 SD | **–** 12 ms | 75% chance of **positive** outcome |
| *Note.* RT = reaction time Since the cutoff score is calculated relative to performance, this is not expected to influence performance of some individuals differently than performance of others. | | | | | | |

In order to emphasize the rewarding and punishing aspects of the task, participants were told that if they performed well on the winning games they could win a prize (i.e., reward). Additionally, they were told that if they did not perform well enough on the losing games they would have to redo the task (i.e., punishment). In order to give the impression that this was checked at the end participants had to write their obtained score of each game on a paper. All participants were told that they performed well enough to not have to redo the task, and they all won a prize (gift bag with a mug, notebook, and pencil).

Following the task, after they were informed that they won the prize and did not have to redo the task, participants answered some questions about the task. To examine whether the reward (winning a prize) and the punishment (redoing the task) were comparable in strength, participants were asked how much they liked that they could win a prize, and how much they disliked that they might had to redo the task. These two questions were answered on a VAS ranging from *Not at all* (0) to *A lot* (100). To examine whether the blue cue becomes a signal of reward, and the red cue a signal of punishment, they were asked how they felt about the blue and the red arrow. These two questions were answered on a VAS ranging from *Very* *negative* (0) to *Very* *positive* (100). To examine whether the blue trials are experienced as more easy than the red trials, they were asked whether it was easy to respond fast enough in blue arrow trials, and red arrow trials. These two questions were answer on a VAS ranging from *Completely disagree* (0) to *Completely agree* (100).

The SOT data were analyzed following Jonker et al., [3]. Facilitated engagement to reward was inferred when participants attend more to rewarding than to non-rewarding cues during the winning games. In other words, when during winning games, they responded faster to targets that appeared in the location preceded by the blue cue than in the location preceded by the red cue. Higher scores reflect more attentional engagement with reward. Facilitated engagement to punishment was inferred when participants attended more to punishing than to non-punishing cues. Thus, when during losing games, they responded faster to targets that appeared in a location preceded by a red cue, than to targets that appeared in a location preceded by a blue cue. Higher scores reflect more attentional engagement with punishment. Difficulty to disengage from reward was inferred when participants had more difficulty to look away from rewarding cues than from non-rewarding cues during winning games. Thus, when they responded slower on uncued blue cue trials, than on uncued red cue trials. Higher scores reflect more difficulty to disengage from reward. Analogously, difficulty to disengage from punishment was inferred when participants had more difficulty to look away from punishing cues than from non-punishing cues during losing games. Thus, when they responded slower on uncued red trials, than on uncued blue trails. Higher scores reflect more difficulty to disengage from punishment (see S2 Table).

| S2 Table. *Calculation of attentional biases to reward and punishment* | | | | | |
| --- | --- | --- | --- | --- | --- |
| **Game** | **Bias** | **Calculation** | **Interpretation** | **Cue delay time** | |
| *Winning* game | Engagement | mean RT cued red trials –mean RT cued blue trials | high score = high AB to reward | 250 ms | Automatic |
|  |  |  |  | 500 ms | Voluntary |
|  | Difficulty to disengage | mean RT uncued blue trials – mean RT uncued red trials | high score = high AB to reward | 250 ms | Automatic |
|  |  |  |  | 500 ms | Voluntary |
| *Losing* game | Engagement | mean RT cued blue trials – mean RT cued red trials | high score = high AB to punishment | 250 ms | Automatic |
|  |  |  |  | 500 ms | Voluntary |
|  | Difficulty to disengage | mean RT uncued red trials – mean RT uncued blue trials | high score = high AB to punishment | 250 ms | Automatic |
|  |  |  |  | 500 ms | Voluntary |
| From *"* Attentional bias for reward and punishment in overweight and obesity: The TRAILS study ", by N.C. Jonker, K.A. Glashouwer, B.D. Ostafin, M.E*.* Van Hemel-Ruiter, F.R.E. Smink, H.W. Hoek and P.J. De Jong, 2016, PLOS ONE, Supplemental Material. Reprinted with permission.  *Note.* RT = reaction time, AB = attentional bias. | | | | | |

SOT response pattern

The general response pattern of the participants was examined (See S3 Table). Paired samples *t*-tests showed a general engagement effect, both on the short and long cue delay trials. Participants were faster on cued blue trials than on cued red trials. On the short cue delay trials a disengagement effect was found, participants had more difficulty to direct their attention away from the blue cues than from the red cues. However, this disengagement effect was not found on the long cue delay trials.

| S3 Table. *Overall differences between blue and red cue trails, separately for different trial types.* | | | | | | |
| --- | --- | --- | --- | --- | --- | --- |
|  |  |  |  | **95 % Confidence interval of the Difference** | |  |
|  |  | **Calculation** | **Cue delay** | **Lower bound** | **Upper bound** | ***p*** |
| WG | **Engagement** | Cued red – Cued blue | Short | 31.66 | 48.59 | <.001 |
|  |  |  | Long | 22.46 | 41.94 | <.001 |
|  | **Difficulty to disengage** | Uncued blue – Uncued red | Short | -26.59 | -1.79 | <.05 |
|  |  |  | Long | -12.32 | 10.67 | .89 |
| LG | **Engagement** | Cued blue – Cued red | Short | -44.90 | -32.00 | <.001 |
|  |  |  | Long | -31.67 | -10.39 | <.001 |
|  | **Difficulty to disengage** | Uncued red – Uncued blue | Short | 15.77 | 44.58 | <.001 |
|  |  |  | Long | -12.31 | 14.52 | .87 |
| *Note. N* = 137, WG = Winning Game, LG = Losing Game. | | | | | | |

SOT task assumptions

Further, we examined the task assumption questions (See S4 Table). In general participants were as positive about that they could win a prize, as they were negative about that they might have to redo the task (*t*(136) = 1.10, *p* = .28). Further, the blue arrow was rated as more positive than the red arrow (*t*(136) = 26.98, *p* < .001), and blue cue trials were rated as more easy than red cue trials (*t*(136) = 11.60, *p* < .001). Since the evaluation of these task aspects might be influenced by individuals’ reward and punishment sensitivity, and we expect group differences on sensitivity to reward and punishment, it was examined whether the AN and CG groups differed in their answers on these questions. This was however not the case.

| S4 Table. *Checking task assumption questions* | |  |  |  |
| --- | --- | --- | --- | --- |
|  | **All**  **(*N* = 137)** | **CG (*n* = 68)** | **AN (*n* = 69)** | **Between-groups test** |
|  | ***Mean (SD)*** | ***Mean (SD)*** | ***Mean (SD)*** | ***t (p)*** |
| How much did you like that you could win a prize | 73.58 (21.86) | 74.21 (22.18) | 72.97 (21.68) | -0.33 (.74) |
| How much did you mind that you might had to redo the task | 70.11 (28.33) | 73.03 (26.75) | 67.23 (29.72) | -1.20 (.23) |
| I think the blue arrow was.. | 76.23 (18.35) | 78.34 (18.21) | 74.16 (18.38) | -1.34 (.18) |
| I think the red arrow was... | 17.00 (13.64) | 17.07 (14.07) | 16.93 (13.31) | -0.06 (.95) |
| It was easy to respond on blue arrow trials | 61.82 (26.54) | 63.50 (28.06) | 60.16 (25.04) | -0.74 (.46) |
| It was easy to respond on red arrow trials | 24.58 (23.03) | 24.13 (22.19) | 25.04 (24.00) | -0.23 (.82) |

Data reduction

Before calculating the attentional bias measures of the SOT, outliers and errors were removed, following van Hemel-Ruiter et al. [4]. First, trials on which participants responded before the target appeared were deleted. For the patients with AN this resulted in the deletion of 7.9% of the trials, and for the healthy controls this was 9.3%. Trials during which participants did not respond whereas they should have responded were also excluded from further analyses. This were 6.9% of the trials in the AN group and 6.3% of the trials in the HC group. Lastly, trials with reaction times below 125 ms (anticipation errors) were deleted. In the AN group this were 7.4% of the trials, and in the HC group 7.9%. No trials with reaction times above 1000 ms (probable distractions) were identified. Mean reaction times and standard deviations per trial type per group can be found in S5 Table.

| S5 Table. *Mean reaction times and standard deviations of the Spatial Orientation Task* | | | | | | | | |
| --- | --- | --- | --- | --- | --- | --- | --- | --- |
|  | **AN (*n*=69)** | | | | **CG (*n*=68)** | | | |
|  | **Cued** | | **Uncued** | | **Cued** | | **Uncued** | |
|  | **Blue** | **Red** | **Blue** | **Red** | **Blue** | **Red** | **Blue** | **Red** |
|  | **Short cue delay time (250 ms)** | | | | | | | |
| WG | 334 (46) | 374 (77) | 499 (94) | 522 (81) | 318 (42) | 359 (51) | 498 (83) | 502 (87) |
| LG | 327 (39) | 360 (54) | 488 (98) | 513 (98) | 321 (35) | 364 (49) | 472 (84) | 507 (98) |
|  | **Long cue delay time (500 ms)** | | | | | | | |
| WG | 364 (73) | 394 (75) | 429 (90) | 418 (83) | 351 (67) | 385 (74) | 407 (82) | 420 (85) |
| LG | 368 (82) | 376 (70) | 429 (103) | 426 (95) | 338 (65) | 371 (64) | 407 (95) | 412 (88) |
| *Note.* CG= Comparison group, AN = Patients with anorexia nervosa, WG = *winning* game, LG = *losing* game. | | | | | | | | |
